# Supplementary material for: Predictive Value of 18F-FDG PET/CT Using Machine Learning for Pathological Response to Neoadjuvant Concurrent Chemoradiotherapy in Patients with Stage III Non-Small Cell Lung Cancer
Source: Cancers (Basel). 2022 Apr 14;14(8):1987. doi: 10.3390/cancers14081987 (PMC9031866; doi:10.3390/cancers14081987)
Supplement: Supplementary file 1 [file cancers-14-01987-s001.zip › Figure S1.pdf]

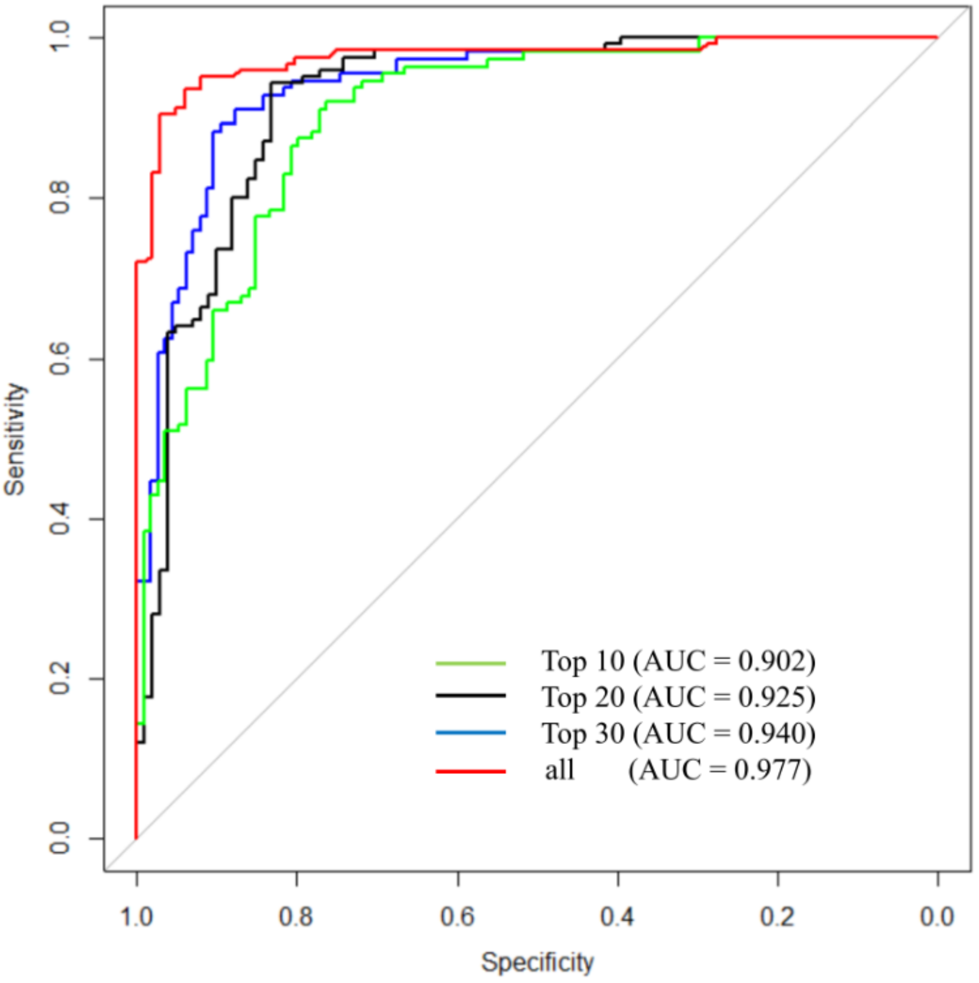

Figure S1. Comparision of ROC curves from random forest according to ranking-based feature selection.
