# Supplementary material for: Predictive Value of 18F-FDG PET/CT Using Machine Learning for Pathological Response to Neoadjuvant Concurrent Chemoradiotherapy in Patients with Stage III Non-Small Cell Lung Cancer
Source: Cancers (Basel). 2022 Apr 14;14(8):1987. doi: 10.3390/cancers14081987 (PMC9031866; doi:10.3390/cancers14081987)
Supplement: Supplementary file 1 [file cancers-14-01987-s001.zip › Table S1.pdf]

Table S1. List of quantitative PET-based radiomic features from CGITA.

Matrix	Index
Cooccurance matrix	Second angular moment, contrast, entropy, homogeneity, dissimilarity, inverse difference moment
Voxel-alignment matrix	Short-run emphasis, long-run emphasis, intensity variability, run-length variability, run percentage, low-intensity run emphasis, high-intensity run emphasis, low-intensity short-run emphasis, high-intensity short-run emphasis, low-intensity long-run emphasis, high-intensity long-run emphasis
Neighborhood intensity difference matrix	Coarseness, contrast, busyness, complexity, strength
Intensity size-zone matrix	Short-zone emphasis, large-zone emphasis, intensity variability, size-zone variability, zone percentage, low-intensity zone emphasis, high-intensity zone emphasis, low-intensity short-zone emphasis, high-intensity short-zone emphasis, low-intensity large-zone emphasis, high-intensity large-zone emphasis
Normalized cooccurance matrix	Second angular moment, contrast, entropy, homogeneity, inverse difference moment, dissimilarity, correlation
Voxel statistics	Minimum SUV, maximum SUV, mean SUV, SUV variance, SUV SD, SUV skewness, SUV kurtosis, SUV skewness (bias corrected), SUV kurtosis (bias corrected), TLG, tumor volume, entropy, SUL_{peak}
Texture spectrum	Max spectrum, black-white symmetry
Texture feature coding	Coarseness, homogeneity, mean convergence
Texture feature coding cooccurance matrix	Second angular moment, contrast, entropy, homogeneity, intensity, inverse difference moment, correlation, variance, code similarity
Neighborhood gray-level dependence	Small-number emphasis, large-number emphasis, number nonuniformity, second moment, entropy

CGITA, Chang-Gung Image Texture Analysis toolbox; SUV, standard uptake value; SD, standard deviation; TLG, total lesion glycolysis; SUL, SUV normalized to lean body mass.
