# Supplementary material for: Predictive Value of 18F-FDG PET/CT Using Machine Learning for Pathological Response to Neoadjuvant Concurrent Chemoradiotherapy in Patients with Stage III Non-Small Cell Lung Cancer
Source: Cancers (Basel). 2022 Apr 14;14(8):1987. doi: 10.3390/cancers14081987 (PMC9031866; doi:10.3390/cancers14081987)
Supplement: Supplementary file 1 [file cancers-14-01987-s001.zip › Table S2.pdf]

Table S2. Predictive performance of random forest according to ranking-based feature selection.

Features	AUC	ACC	F1	Precision	Recall
Top 10	0.902 [*,†]	0.823 [*,†,‡]	0.837 [*,†,‡]	0.769 [*,†,‡]	0.920
Top 20	0.925 [‡]	0.867 [*,§]	0.876 [*,§]	0.815 [*,§¶]	0.940
Top 30	0.940 [*]	0.881 [†¶]	0.897 [†¶]	0.855 [†,§,#]	0.942
All	0.977 [†,‡]	0.934 [‡,§¶]	0.940 [‡,§¶]	0.937 [‡,#]	0.944

AUC, area under curve; ACC, accuracy; *, †, ‡, §, ¶, #, $p < 0.05$.
